# Cellulose-Based Conductive Materials for Energy and Sensing Applications

**DOI:** 10.3390/polym15204159

**Published:** 2023-10-19

**Authors:** Duan-Chao Wang, Sheng-Nan Lei, Shenjie Zhong, Xuedong Xiao, Qing-Hui Guo

**Affiliations:** 1 Stoddart Institute of Molecular Science, Department of Chemistry, Zhejiang University, Hangzhou 310027, China; 2 Hangzhou Global Scientific and Technological Innovation Center, Zhejiang University, Hangzhou 311215, China; 3 Hangzhou Institute of Technology, Xidian University, Hangzhou 311231, China

**Keywords:** cellulose, bioeconomy, conductive materials, batteries, supercapacitors, sensors

## Abstract

Cellulose-based conductive materials (CCMs) have emerged as a promising class of materials with various applications in energy and sensing. This review provides a comprehensive overview of the synthesis methods and properties of CCMs and their applications in batteries, supercapacitors, chemical sensors, biosensors, and mechanical sensors. Derived from renewable resources, cellulose serves as a scaffold for integrating conductive additives such as carbon nanotubes (CNTs), graphene, metal particles, metal–organic frameworks (MOFs), carbides and nitrides of transition metals (MXene), and conductive polymers. This combination results in materials with excellent electrical conductivity while retaining the eco-friendliness and biocompatibility of cellulose. In the field of energy storage, CCMs show great potential for batteries and supercapacitors due to their high surface area, excellent mechanical strength, tunable chemistry, and high porosity. Their flexibility makes them ideal for wearable and flexible electronics, contributing to advances in portable energy storage and electronic integration into various substrates. In addition, CCMs play a key role in sensing applications. Their biocompatibility allows for the development of implantable biosensors and biodegradable environmental sensors to meet the growing demand for health and environmental monitoring. Looking to the future, this review emphasizes the need for scalable synthetic methods, improved mechanical and thermal properties, and exploration of novel cellulose sources and modifications. Continued innovation in CCMs promises to revolutionize sustainable energy storage and sensing technologies, providing environmentally friendly solutions to pressing global challenges.

## 1. Introduction

In modern society, the importance of energy and sensing applications cannot be overemphasized. These two fundamental pillars play a key role in shaping our connected world, driving innovation and improving our quality of life. Energy powers machines and devices in all forms, powering everything from our homes and transportation systems to the vast array of electronic devices that have become integral to our daily lives [1]. In addition, sensing applications have revolutionized the way we interact with our environment. From smartphones equipped with a plethora of sensors to sophisticated industrial equipment, sensing technologies provide us with valuable insights into our surroundings, enabling efficient resource management, enhanced safety measures, and advanced healthcare solutions [2]. The synergy between energy and sensing applications not only underpins industrial growth but also enables the development of sustainable, environmentally friendly solutions to some of the most pressing global challenges, such as climate change and resource scarcity [3,4]. In this context of technological advancement, the development of next-generation energy and sensing materials using sustainable cellulose will be a critical research direction.

In recent years, the search for sustainable and environmentally friendly materials has become a primary focus of science and industry, and the quest for innovative materials that harmonize cutting-edge performance with ecological awareness has reached new heights [5,6]. The growing global demand for energy storage, power generation, and sensing technologies has increased the need for materials that not only fulfill performance requirements but also comply with sustainability principles [7]. It is in this context that cellulose-based conductive materials (CCMs) have emerged as a highly promising class of materials that are expected to play a key role in redefining energy and sensing applications [8].

Cellulose is a naturally abundant polymer found in plant cell walls derived from renewable resources such as wood, cotton, and agricultural residues, which makes it one of the most ubiquitous organic compounds on the planet. It is the structural backbone of plant growth, contributing to the sturdiness of trees, the flexibility of cotton fibers, and the resilience of agricultural residues [8,9,10,11,12,13,14]. Historically, cellulose has been used primarily in the paper and textile industries due to its superior mechanical properties and biocompatibility [15]. One fundamental property of cellulose has prevented its widespread use in energy and sensing materials: cellulose is essentially an insulator. This limitation then stimulated the development of CCMs, materials that cleverly combine renewable cellulose with electronic conductivity [11,13,16,17], thus opening new avenues for innovation and sustainability in materials science.

The importance of CCMs lies in their sustainability and their diverse combination of properties, which can synergistically enhance their suitability for a wide range of applications [2,18,19]. Firstly, cellulose itself can be transformed from an insulator to a conductor with carbonization [20,21,22], and cellulose can be pre-formed and retain a certain structure after carbonization for use in energy and sensing materials [23,24]. Another one is the composite strategy; composite materials are usually realized by chemically modifying cellulose or by incorporating conductive additives and offer three main advantages [18,25], electrical conductivity, mechanical flexibility, and partial degradability, making them well suited for a wide range of applications. In addition, their potential for customization and functionalization opens the door to innovative solutions in the fields of energy storage and sensing.

**Figure 1:**
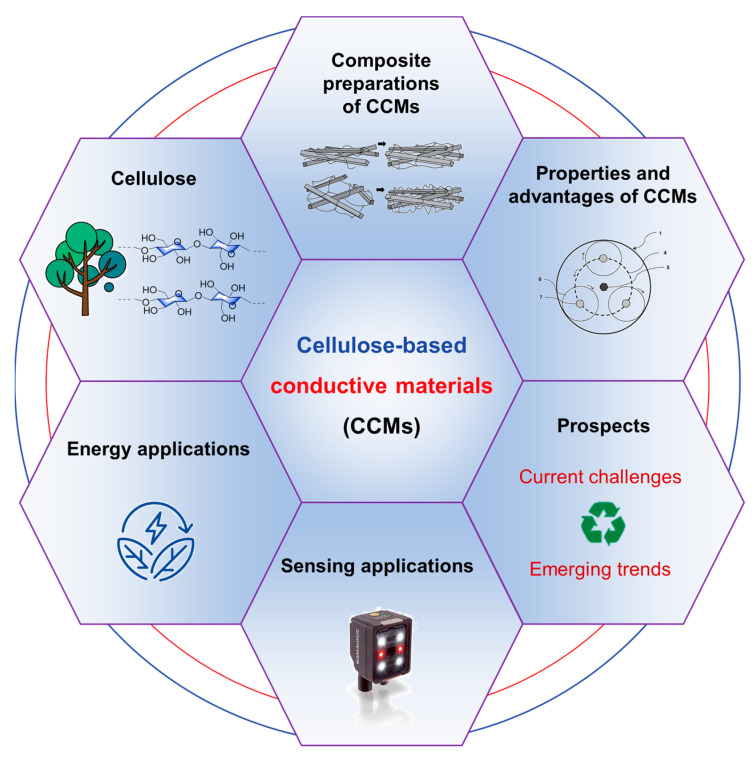
Schematic diagram of the topics on cellulose-based conductive materials (CCMs) in this review.

In this review, we provide a comprehensive summary of the last five years of research on CCMs, dissecting their synthesis techniques, structural properties, advantageous properties, and what makes them outstanding for energy and sensing applications (Figure 1). This review will delve into the potential of CCMs in the foreground of energy applications, including batteries and supercapacitors, each of which provides a unique platform for exploiting the distinctive properties of CCMs. In addition, we will reveal their role as specialized sensing materials for applications such as chemical and mechanical sensing, where their flexibility and responsiveness can find multiple innovative applications. It is hoped that the chapters will illustrate the current advances in materials science and future needs, such as scalability, long-term stability, and cost-effectiveness, brought about by further aspects of the synthesis and utilization of these polymers. The aim of this work is to demonstrate how CCMs not only embody the principles of sustainability but also have the potential to re-think the modern technological landscape, propelling us towards a greener and technologically advanced future. Indeed, these materials may hold the key to solving some of the most pressing problems of our time, from sustainable energy to rapid environmental monitoring, and stimulate our imagination as we contemplate a world where green materials catalyze modern technological revolutions, guiding us towards a future where sustainability and innovation go hand in hand.

## 2. Cellulose and Nanocellulose

**Figure 2:**
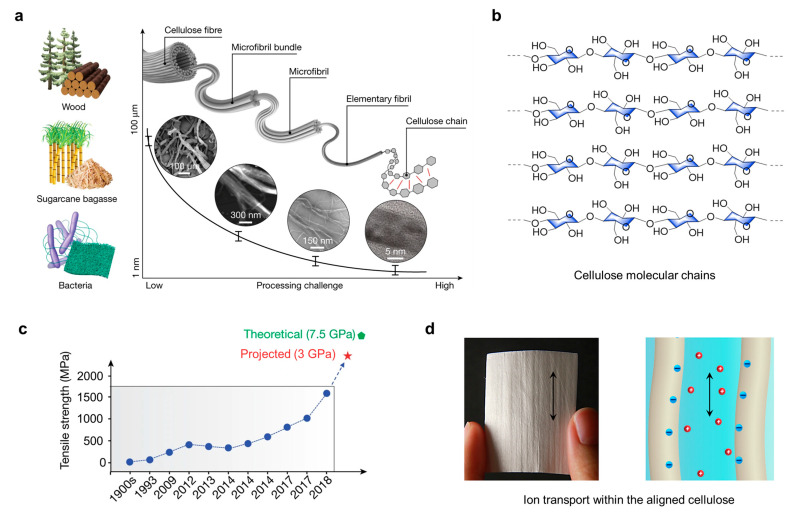
(**a**) Sources of cellulose and its multiscale hierarchical structure. (**b**) Cellulose molecular chains. (**c**) Mechanical property enhancement timeline of cellulose structures. Reprinted with permission from ref. [8]. (**d**) Schematic representation of bulk cellulose materials and their ion transport properties. Reprinted with permission from ref. [34].

Cellulose and nanocellulose are two important materials that have attracted much attention in the field of materials science [26,27,28,29]. Nanocellulose can be extracted from bulk cellulosic materials by means of hydrolysis and other means. Cellulose exhibits different properties at the bulk material scale and nanoscale. As shown in Figure 2a, cellulose from biomass and bacteria can be decomposed into protofibrils of decreasing diameter, with a size distribution that decreases from 100 to 2–4 nm, and ultimately stripped down to chains of cellulose molecules with supramolecular structures (Figure 2b). Cellulose is a natural organic compound found in plant cell walls. It is a polysaccharide composed of glucose molecules that form a fibrous structure through hydrogen bonding and other interactions. Cellulose plays the role of supporting and protecting cells in plants and therefore has excellent mechanical properties. In nearly three decades of research on cellulose, the mechanical properties of cellulosic materials have been taken to new heights through the structural design of multiscale cellulose (Figure 2c) [8,30,31,32]. For example, the mechanical tensile strength of films made only from raw nanocellulose fibers can reach about 300–500 MPa, which is much higher than that of conventional paper made from loose cellulose. Based on the excellent mechanical properties of cellulose, it is also widely used in textiles, biology, energy, sensing [8], and biocomposites [33]. Nanocellulose materials, on the other hand, are materials developed on the basis of cellulose and have the molecular structure of cellulose at the microscopic scale (Figure 2a).

Their preparation usually involves mechanical or chemical production and processing techniques. These materials are highly valued for their unique properties. Cellulose nanomaterials have a high specific surface area, excellent mechanical strength, outstanding transparency, and biocompatibility; these properties make them promising for a wide range of applications. Driven by nanotechnology and materials science, nanocellulose materials have been used in the preparation of cellulose films, cellulose fibers, drug delivery carriers, biomedical materials, and so on. The specific surface area of nanocellulose itself was measured using the Brunauer–Emmett–Teller (BET) method to be 1.29 ± 0.10 m^2^/g [35]. If nanocellulose is prepared as an aerogel material, the specific surface area of cellulose-based aerogels is as high as 600 m^2^/g [36]. These materials have great potential in environmental, biomedical, energy, and sensing fields, among others, as they are not only sustainably produced but reduce the negative impact on the environment. Through physical or chemical methods, cellulose and nanocellulose are highly tunable in terms of their morphology, size, and surface chemistry, allowing for unique mechanical, optical, thermal, fluidic, and ionic properties (Figure 2d) [34].

Nanocellulose can be subdivided into cellulose nanofibers, cellulose nanocrystals, and bacterial cellulose. They have similar chemical compositions but differentiated structures for different applications. These cellulosic materials are all characterized by sustainability, biodegradability, and renewable resources and therefore have a potentially important role to play in replacing traditional materials.

**Figure 3:**
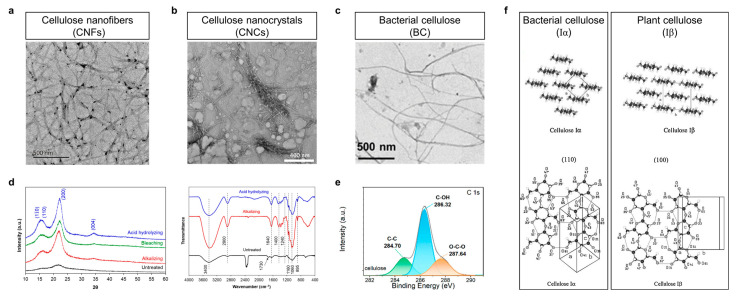
(**a**) Transmission electron microscope (TEM) image of CNFs. Reprinted with permission from ref. [37]. (**b**) TEM image of CNCs. Reprinted with permission from ref. [45]. (**c**) TEM image of BC. Reprinted with permission from ref. [50]. (**d**) X-ray diffraction (XRD) patterns and Fourier-transform infrared (FTIR) spectra of cellulose. Reprinted with permission from ref. [48]. (**e**) X-ray photoelectron spectroscopy (XPS) C 1s spectrum of cellulose. Reprinted with permission from ref. [49]. (**f**) Schematic representation of the different cellulose I crystal types; BC is generally Iα, while plant cellulose is generally Iβ. Reprinted with permission from ref. [53].

Cellulose nanofiber (CNF) is a nanoscale fiber extracted from natural cellulose sources and is prepared with mechanical shearing, acid hydrolysis, or ball milling. The size of a CNF is characterized at the nanoscale with a high specific surface area and fibrous structure, usually with diameters between tens and hundreds of nanometers and lengths of up to a few micrometers (Figure 3a) [37]. CNF is outstanding in terms of sustainability and biodegradability and can be used to prepare biodegradable packaging materials, high-strength pulp, transparent films, biomedical engineering materials, nanocomposites, food packaging, and stabilizers [38,39,40,41]. Its renewability, high strength, and environmentally friendly properties make it one of the key materials for replacing traditional materials and promoting sustainable development. CNF composites with polymers have a very wide range of prospects, which can help to reduce plastic pollution, improve material performance, reduce energy consumption, and promote sustainable development and therefore have a great potential in various industrial fields [40,42,43,44], which is expected to improve the environment and reduce carbon footprint, while providing innovative material solutions.

Cellulose is associated with alternating crystalline and amorphous regions, and when the amorphous regions are removed, highly crystalline cellulose nanocrystals (CNCs) can be obtained. The length and diameter of CNCs are smaller than those of CNF, which is generally several hundred nanometers in length and a few tens of nanometers in diameter (Figure 3b) [45]. The method of CNC preparation usually consists of the following steps: bulky cellulose is usually chemically or biologically pre-treated to remove the non-cellulosic components, such as hemicellulose and lignin; cellulose undergoes acid hydrolysis to break it down into CNCs; commonly used acids include sulfuric acid and hydrochloric acid; after acid hydrolysis, CNCs need to be separated and purified with washing and centrifugation, etc.; finally, the CNCs are usually prepared into powders using methods such as freeze-drying or vacuum-drying. The size of CNCs is at the nanometer level, and they have a higher specific surface area than CNF. They also have a high degree of crystallinity, giving them excellent mechanical strength and hardness. At the same time, CNCs remain renewable, environmentally friendly, and biocompatible. In particular, CNCs have good transparency and can be used to prepare transparent nanocomposites. Therefore, CNC composites with polymers have a wide range of applications in many fields, including food packaging, drug delivery, pulp and paper improvement, and biomedical materials [46,47]. They can improve the mechanical properties, moisture barrier properties, and biodegradation of polymer materials.

Cellulose and nanocellulose show similar characteristics in various spectra; in other words, spectroscopy is the most convenient way to identify them. The X-ray diffraction (XRD) patterns and Fourier-transform infrared (FTIR) spectra of CNCs are shown in Figure 3d [48]. The characteristic peaks at 14.9°, 16.5°, 22.8°, and 34.1° in the XRD patterns correspond to the (1–10), (110), (200), and (004) crystal planes of the natural cellulose, respectively, and both the bulky cellulose and the nanofibers of cellulose obtained with different methods exhibited these features. Differently, the FTIR spectra of untreated bulk cellulose feedstock and the obtained cellulose differ from each other. The characteristics of the un-removed hemicellulose and lignin in the bulk cellulose appeared, which gradually disappeared after alkalization or even completely disappeared after acid digestion. The spectrum at 1730 cm^−1^ shows acetyl and ester groups from hemicellulose or carboxylic groups from lignin, and the spectrum at 1240 cm^−1^ shows out-of-plane vibrations from aryl groups. In contrast, the characteristic peaks of nanocellulose in the FTIR spectra are the O–H stretching vibration at 3400 cm^−1^, the C–H tensile vibration at 2900 cm^−1^, and the H–C–H and O–C–H internal bending vibration at 1400 cm^−1^. X-ray photoelectron spectroscopy (XPS) [49] in Figure 3e shows the different bonding in the cellulose molecule regarding the C atoms around the cellulose, which has three peaks at 284.70, 286.32, and 287.64 eV, corresponding to C–C, C–OH, and O–C–O bonds, respectively. Overall, subclasses of cellulose can be quickly identified and discriminated using electron microscopy and the three spectra mentioned above.

Bacterial cellulose (BC) has a similar chemical structure to plant cellulose, but it is a biopolymer produced by bacteria and synthesized by strains such as *Acetobacter xylinum*. These bacteria synthesize cellulose using a carbon source such as glucose under appropriate conditions and externally secrete it around the cell to form a fibrous structure. The chemical structure of BC is similar in size to that of CNFs (Figure 3c) [50], which are linear polysaccharides consisting of glucose molecules linked by β-1,4-glycosidic bonds. This similarity allows BC to be used as a substitute for plant cellulose in some applications, and it has excellent physicochemical properties, including high purity, high strength, high specific surface area, and water retention capacity. It has a number of characteristics, such as transparency, flexibility, temperature resistance, and biocompatibility, which make it uniquely suited to a variety of applications. For example, BC can be used in the food industry, e.g., as stabilizers, thickeners, and humectants, and in the production of textiles to increase the softness and water absorbency of fabrics [51,52]. The production of BC is often more sustainable as it can be sourced from discarded agricultural wastes or industrial by-products without the need for large amounts of land and water resources. It is worth noting that the crystal structure of BC is of the fibrillar Iα crystalline type, whereas plant-derived fibrils are generally of the Iβ crystalline type, as in Figure 3f. Only minor differences were observed between fibrils Iα and Iβ. Cellulose Iβ forms a two-chain monoclinic unit cell of space group P21, whereas Iα crystallizes in a one-chain triclinic unit cell P1 and mostly occurs combined with Iβ in the fibrils. Cellulose Iα exhibits fibrillar disaccharides as the structural unit, and cellulose Iβ exhibits two symmetrically related glucans as the fibrillar orientation c structural unit [53].

## 3. Preparation of Cellulose-Based Conductive Materials (CCMs)

Cellulose has a natural advantage in the preparation of advanced flexible energy devices and sensors because of its rich functional groups, unique network and pore structure, high flexibility, and low coefficient of thermal expansion [13]. However, before application, cellulose needs to be carbonized or compounded with conductive substances to match the needs of energy and sensor devices. Carbonized or composited cellulose/nanocellulose with conductive materials will bring electrical conductivity, which is an important step towards building on their excellent properties and enabling them to shine in the energy and sensing fields. While pre-treated bulk cellulose can be cut into desired forms and carbonized or composited with conductive materials to fabricate conductive materials, nanocellulose needs to be pre-formed before subsequent processes. This, coupled with the design of composite structures with some polymers, can assist in the shaping and stabilization of carbonized cellulose and its composite conductive structures [54,55]. Regarding the preparation of CCMs, they can be divided into the following three strategies: carbonized cellulose, cellulose composite carbon materials, cellulose composite metal particles, and cellulose composite conductive polymers. Cellulose is primarily used to disperse nano-conductive materials, polymers are able to act as a stabilizing substrate (cellulose, itself a polymer, also provides this function), and added conductive matters both assist in molding and provide conductive pathways; sometimes, these strategies are mixed.

### 3.1. Carbonized Cellulose Materials

Bulk cellulose, CNF, and BC are used more in the carbonization process application because they are still able to maintain their unique network and pore size structure during the carbonization process [56,57]. Carbonized cellulose is natural cellulose that has been processed through high-temperature pyrolysis ovens or high-pressure reactors, etc., during which most of the non-carbon elements are removed. Carbonized cellulose still maintains a high porosity and high specific surface area and provides electrical conductivity.

**Figure 4:**
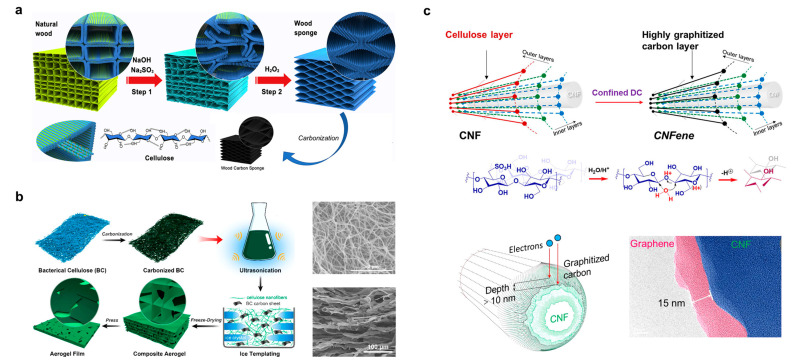
(**a**) Elastic carbon material of natural wood origin. Reprinted with permission from ref. [58]. (**b**) Carbonized BC with wood-sourced CNF composite aerogel. Reprinted with permission from ref. [24]. (**c**) Conductive CNFene carbonized at low temperature and atmospheric pressure. Reprinted with permission from ref. [20].

Chen and Hu et al. fabricated lightweight and highly compressible wood-derived carbon sponges directly from natural balsa wood through a top-down approach [58], as shown in Figure 4a. Lignin and hemicellulose were first removed from the cell walls of the wood using a chemical treatment and then converted into a spring-like compressible layered structure using pyrolysis. The conductivity of the final samples was 0.04 to 1.66 S cm^−1^. If this carbon sponge with a lamellar structure is used to compound elastic polymer materials, it can be used for mechanically responsive materials.

Chen et al. combined carbonized BC with excellent electrical conductivity with wood-derived CNF and prepared aerogels with porous structures and aligned channels using oriented ice templates and freeze-drying [24]. Figure 4b illustrates the fabrication of this carbonized BC/CNF composite aerogel film, where the carbonized BC serves as the conductive pathway while the CNF serves as the natural polymer template. It can be seen that the wood fibers constructed from CNF bundles have a hierarchical structure, with homogeneous CNF ranging from 21 to 47 nm in diameter and approximately 374 ± 245 nm in length. After high-temperature pyrolysis of the BC film, the obtained carbonized BC film was added to the CNF suspension, and the mixture was subsequently sonicated. The composite conductive aerogel films were finally prepared using directional freeze-drying and compression. Scanning electron microscope (SEM) images show that the composite is expected to have well-arranged pore arrays due to the targeted ice templates, as well as randomly distributed carbonized BC flakes in contact with each other after compression.

Figure 4c demonstrates a unique carbonization process at a low temperature for cellulose, where Wang et al. surprisingly obtained conductive CNF (CNFene) with a carbon layer of about 10 nm by heating it up to 90 °C directly after extracting CNFs using dilute sulfuric acid [20]. Sulfuric acid can be used as a hydrolysis reagent for the extraction of nanocellulose and as an initiator for dehydration and carbonization at slightly higher temperatures, thus obtaining carbonized CNF in one step. This new method eliminates the bulky and energy-consuming muffle furnaces and decreases the temperature of the cellulose carbonization from 800 °C to 90 °C, with a reduction in energy consumption of more than 80%/g. A variety of biomasses were prepared for the low-temperature carbonization of cellulose in this study, and the highest conductivity of the final samples reached 1.1 S/cm. This method is expected to open up a new way to carbonize cellulose in a more sustainable manner.

### 3.2. Cellulose Composite Carbon Materials

Carbon materials are very popular in current research, especially low-dimensional carbon materials, such as graphene and carbon nanotubes (CNTs). They have excellent electrical and thermal conductivity and can produce a variety of quantum effects [59,60]. Cellulose composite carbon materials can inherit the excellent properties of carbon materials and at the same time solve the problem of low-dimensional carbon materials being difficult to build engineering materials with. Combining the advantages of both, cellulose composite carbon materials can be prepared for flexible and portable energy and sensor devices [13].

**Figure 5:**
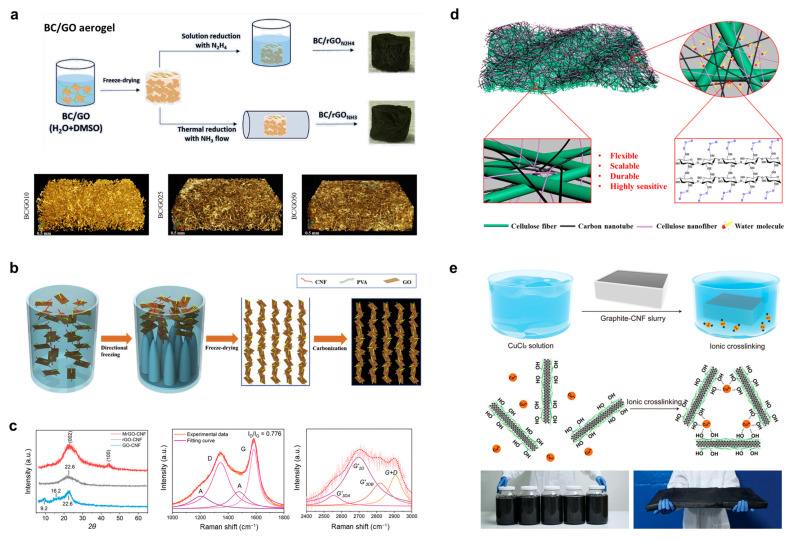
(**a**) Preparation of BC/graphene oxide (GO) aerogels. Reprinted with permission from ref. [61]. (**b**) Graphene/PVA/CNF composite aerogel prepared using a directional freezing and carbonization process. Reprinted with permission from ref. [62]. (**c**) XRD and Raman spectra of CNF/graphene composites before and after microwave treatment. Reprinted with permission from ref. [63]. (**d**) A CNF/CNT composite conductive bilayer paper prepared using a spraying process. Reprinted with permission from ref. [64]. (**e**) Preparation process of graphite flake/CNF foam crosslinked by Cu^2+^ ions. Reprinted with permission from ref. [65].

Pinto et al. prepared a multifunctional BC/graphene oxide (GO) aerogel [61], as shown in Figure 5a, and they modulated the lyophilized aerogel’s porous structure. Crosslinking between different nanophases was also induced by reducing the aerogel through ammonia heat treatment, and the BC/GO aerogels obtained were highly thermally stable with a maximum conductivity of 8.7 × 10^−1^ S/m. These sustainable BC/GO aerogels can be used in fields such as packaging, atmospheric and water treatment, or energy. Zhou et al. developed a graphene/polyvinyl alcohol (PVA)/CNF composite nanofibrillar cellulosic carbon aerogel (Figure 5b) prepared using a directional freezing and carbonization process [62], which has a low density of 6.17 mg/cm^3^ and a high porosity of 99.61%. The cellulose carbon aerogel is anisotropic, hydrophobic, and lipophilic, and its layered interpenetrating three-dimensional (3D) porous structure, good compression recovery, and thermal stability endowed it with a high adsorption capacity (up to 288 times its own weight) and recycling ability. Shi et al. proposed a CNF composite–reduced graphene oxide (RGO) strategy for the preparation of an anode in sodium-ion (Na-ion) batteries using microwave heating. The composite electrode was rapidly carbonized, and Na was added using microwave heating, and the capacity of 340 mAh/g was still maintained after 200 charge–discharge cycles. The ingenuity of this strategy lies in the fact that the small amount of RGO added can be used as an initiator for the ultra-high heating rate of microblogging, while the chemically modified CNF is the main carrier of Na ions, which synergistically improves the electrochemical performance of Na-ion batteries. Figure 5c shows the XRD and Raman spectra of CNF/graphene composites before and after the microwave treatment in this study [63], where the characteristics of the CNF are weakened while the interlayer spacing reaches 0.39 nm and the proportion of sp^2^ carbon changes. The charge transfer resistance of the samples prepared in this study was as low as 48 Ω, much lower than that of commercial carbon. Zhu et al. used a spraying process to prepare a CNF/CNT composite bilayer paper sensor (Figure 5d) [64], where the paper is a typical product of cellulose fiber. The CNF in the micro–nano-layered structure on the surface of this sensor can rapidly exchange water molecules with the external environment through the surface hydroxyl groups, while the CNT is responsible for transmitting the response electrical signals with a maximum current response value of 65%.

Graphite flakes can also be composited with cellulose to prepare foams. Chen et al. developed a graphite sheet/CNF foam with expandable Cu^2+^ ion crosslinking without lyophilization (Figure 5e) [65]. The manufacture of this foam is free of petroleum-based raw materials and low-energy and -cost. The foam maintains mechanical robustness in the wet state, has a low thermal conductivity of 0.05 W/mK, and is also recyclable and degradable. Graphite flake/CNF foam is a sustainable alternative to “white pollution (high molecular weight plastic pollution from the petrochemical industry)”.

### 3.3. Cellulose Composite Metal Particles and Inorganic Compounds

The composite preparation of metal/inorganic compounds such as metal particles, metal oxide particles, liquid metals, and MXene with cellulose has also attracted more and more attention, based on which researchers have developed a variety of effective conductive composite strategies. High conductivity means low electrical resistance of the material, which will result in low power consumption and heat generation in energy and sensing applications, as well as more efficient conduction of electrical signals.

**Figure 6:**
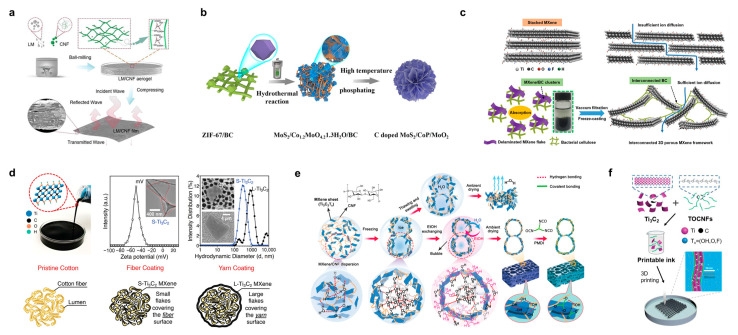
(**a**) Preparation method for self-supported liquid metal/CNF composite films and their structures. Reprinted with permission from ref. [69]. (**b**) Preparation of BC-loaded ternary hetero-structured nanoflower composite flower structures. Reprinted with permission from ref. [70]. (**c**) Microscopic assembly mechanism of BC/MXene-composed 3D porous films. Reprinted with permission from ref. [72]. (**d**) Physicochemical properties and schematic microstructure of knittable and washable cellulose/MXene yarns. Reprinted with permission from ref. [73]. (**e**) Schematic preparation mechanism of CNF/MXene composite aerogel with bionic pearl layer structure. Reprinted with permission from ref. [74]. (**f**) MXene/CNF composite ink for 3D printing of smart fibers and textiles. Reprinted with permission from ref. [75].

A self-supporting cellulose/RGO/silver (Ag) nanoparticle composite membrane was prepared by Zou et al. [66]. Cellulose filter paper, GO, and silver ammonia complexes were first prepared into a slurry, silver ammonia was reduced to Ag nanoparticles under heat treatment, and a thin film was prepared using vacuum filtration; then, hot hydrazine vapors were used to reduce GO to RGO, and the final vimentin/RGO/Ag nanoparticle composite film was obtained, which had a thickness of about 400 μm and a low electrical resistivity as low as 0.17 Ω/sq. Fei et al. chose a metal–organic framework (MOF) as the source of metal particles, and ZIF-67 was chosen to be anchored on CNF, and the composite structure was annealed to obtain a cobalt/carbon@CNF aerogel [67], which showed an excellent electromagnetic shielding effect. The conductivity of the sample was increased to 2.35 S/m, and the specific shielding effectiveness reached 20,172.4 dB cm^3^/g after the sample was treated at 900 °C for 3 h in an argon atmosphere. Garino et al. used a microwave method to synthesize RGO/SnO_2_ and assembled it with micro-fibrillated cellulose to obtain a nanocomposite membrane with good toughness and ductility [68]. The composite films showed excellent performance in capacitive behavior and catalytic redox reactions. The maximum capacitance value of the composite membrane was 53 F/g at a scan rate of 10 mV/s. Liao et al. proposed a strategy for the composite of liquid metal and cellulose (Figure 6a) [69]. In this study, self-supported liquid metal/CNF composite films with a thickness of 4 μm were prepared using processing strategies such as ball milling and dispersion, freeze drying, and compression molding. Ball milling crushed the oxide shells of the liquid metal, and the CNF formed a continuous conductive pathway with an impressive conductivity of 96,000 S/m and a tensile strength of more than 30 MPa. Wang et al. used a hydrothermal reaction and high-temperature phosphatization to prepare a BC-loaded ternary heterostructure nanoflower composite flower composite structure (Figure 6b) [70], which was able to be used for the hydrogen precipitation reaction in a wide pH range. In HER tests, the best sample achieved an initial overpotential of 27 mV in 1.0 M KOH and current densities of 10 mA/cm^2^ at overpotentials of 123.4, 150, and 139 mV in 0.5 M H_2_SO_4_, 0.1 M PBS, and 1.0 M KOH, respectively. The alkaline two-electrode device consisting of MoS_2_/CoP/MoO_2_ doped with carbon delivered 10 mA/cm^2^ at a low potential of 1.51 V and provided a current of 10 mA/cm^2^.

MXene is an emerging material with a high electrical conductivity (2 × 10^5^ S/m), large specific surface area, rich surface chemistry, and easy dispersion in many solvents, including water. Most importantly, it has a special two-dimensional structure that confers excellent electrochemical properties that can be used for energy storage applications, including the ability to transport ions rapidly, a pseudocapacitive charge storage mechanism with double-electric-layer behavior, and mechanical equilibrium at the nanoscale level that can accommodate larger radius ions, such as lithium ions [71]. Cellulose can be used as a molding framework for MXene to fabricate 3D self-supported composites. Wang et al. reported that BC/MXene formed 3D porous films, as shown in Figure 6c [72]. The MXene auto-absorbed and dispersed in a highly interconnected network of BC, which formed a continuous electron and particle transport channel for electrode materials in flexible supercapacitors. The BC/MXene composite electrodes have mass and area capacitances as high as 416 F/g and 2084 mF/cm^2^, respectively. In this study, a flexible asymmetric pseudocapacitor was fabricated by combining a polyaniline (PANI)/BC cathode with a BC/MXene anode in an acidic electrolyte. This aqueous asymmetric supercapacitor has a high area capacitance of up to 925 mF/cm^2^. Uzun et al. developed a knittable and washable cellulose/MXene yarn, as shown in Figure 6d, which is highly conductive and can be used to fabricate wearable devices on an industrial scale [73]. The composite yarn has an MXene loading of 77 wt.% and a conductivity of 440 S/cm and can withstand about 80 washing cycles, which builds a platform for smart textiles. Wu et al. prepared a highly conductive, lightweight, robust, and large-area aerogel composite of CNF and MXene, which is characterized by a biomimetic pearl-layered structure formed by using the strong interaction between CNF and MXene; high mechanical lightness and stability were maintained (Figure 6e), and this robust structure was such that this composite aerogel could be prepared dry under ambient pressure [74]. At a polymer content of 80 wt.%, the electrical conductivity of the CNF/MXene aerogel dried at atmospheric pressure was 1.8 S/m. The CNF/MXene aerogels demonstrated advantages in electromagnetic shielding, light-to-heat conversion, and oil-absorbent applications. In particular, due to the micron-sized pores created by CNF/MXene, these aerogels have achieved electromagnetic interference (EMI) shielding of 42 to 81 dB at densities of only 10 to 45 mg/cm^3^. Cao et al. developed an MXene-reinforced CNF ink for the 3D printing of smart fibers and textiles, as shown in Figure 6f [75]. The study was carried out in 2,2,6,6-tetramethylpiperidine-1-oxylradi-cal (TEMPO) blended with oxidized CNF and titanium-based MXene to obtain a printing ink with good rheological properties. The CNF/MXene fibers obtained from printing had a structure similar to that of natural plant fibers and possessed stimulus responsiveness induced by a variety of factors. The conductivity of the composite fibers exhibited a significant enhancement with the increase in the content of MXene (Ti_3_C_2_) nanosheets, reaching a maximum conductivity of 211.0 S/m.

### 3.4. Cellulose Composite Conductive Polymers

**Figure 7:**
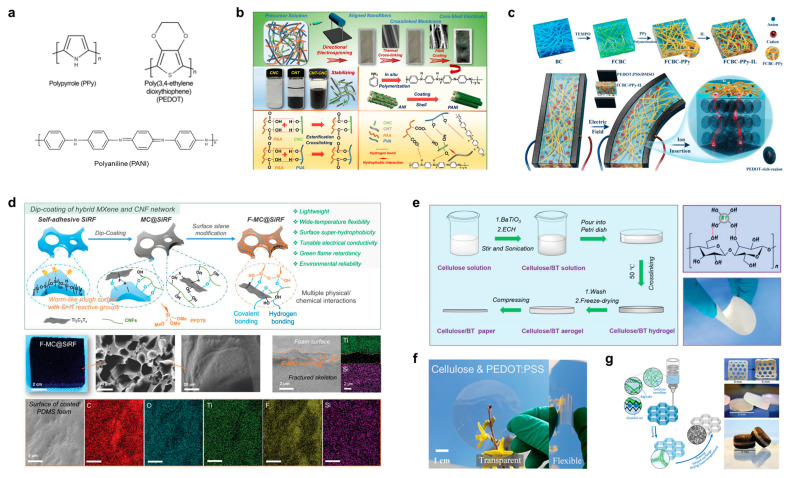
(**a**) Molecular formulae of PPy, PEDOT, and PANI. (**b**) Preparation of CNC-CNT/PVA-PAA composite fiber membrane and schematic diagram of its composite structure. Reprinted with permission from ref. [79]. (**c**) Preparation of BC-PPy-IL composite artificial muscle and its structural schematic. Reprinted with permission from ref. [80]. (**d**) PDMS-loaded CNF/MXene self-adhesive material and its physicochemical characterizations. Reprinted with permission from ref. [81]. (**e**) TENG materials assembled from porous cellulose paper/BaTiO_3_/PDMS. Reprinted with permission from ref. [82]. (**f**) Schematic of the preparation mechanism of regenerated cellulose/AgNWs/PEDOT:PSS composite conductive films. Reprinted with permission from ref. [83]. (**g**) Composite highly conductive aerogels prepared from CNF, alginate, and PEDOT:TOS using 3D printing. Reprinted with permission from ref. [84].

Conductive polymers are electrically conductive materials with conjugated main electrons in the main chain of the molecule, which can either be electrically conductive themselves or doped with other materials that are also electrically conductive [76]. Conductive polymers can also be filled with composite materials [77], with surface mixing or lamination of ordinary polymer materials and a variety of conductive materials to obtain conductivity; conductivity can reach more than 1000 S/cm of polymer material. Currently, conductive polymers such as polyacetylene (PA), polythiophene (PT), polyaniline (PANI), polypyrrole (PPy), and poly(3,4-ethylenedioxythiophene) (PEDOT) polymers are being added to cellulose to obtain flexible composite conductive materials with 3D structures such as fibers, films, and gels [78]. Recently used as composites with cellulose are PANI, PPy, and PEDOT with molecular formulae, commonly, as shown in Figure 7a; other nanoparticles are also added to the composite system to bridge the gaps and discontinuous regions of the cellulose/conducting polymer composite, and elastomeric polymers have also been used as the supporting substrate.

With the help of PVA and polyacrylic acid (PAA), Han et al. labeled a CNC-CNT/PVA-PAA composite fibrous membrane using a classical spinning technique, and then PANI was in situ synthesized on this membrane to obtain a composite conductive membrane (Figure 7b) [79]. The in situ grown PANI was spread out to form a shell on the surface of the fibers, and the whole membrane exhibited a large porosity and high specific surface area. The tensile strength of the composite conductive membrane reached 54.8 MPa, and the conductivity was 0.44 S/m. Wang et al. developed an artificial muscle based on carbonylated BC and PPy [80], as shown in Figure 7c. Porous and conductive BC-PPy nanocomposites were prepared by chemically oxidizing and polymerizing BC surfaces in an aqueous medium. The BC-PPy-IL (1-ethyl-3-methylimidazolium tetrafluoroborate, [EMIM][BF_4_]) nano-biocomposite membrane was further synthesized by fast-drying casting of BC-PPy with IL. The designed membranes exhibited remarkable electrochemical properties, a simple and fast charge transfer response, and tuned mechanical properties. Finally, ionic actuators were prepared by depositing dimethyl sulfoxide (DMSO)-treated PEDOT-polystyrene sulfonate (PEDOT:PSS) on both sides of the proposed membranes.

A PDMS-loaded CNF/MXene self-adhesive material was prepared using a silicone foaming and impregnation strategy by Chen et al. [81]. The presence of considerable oxygen groups in CNF and MXene allowed them to be impregnated into porous PDMS foams under ultrasonication and vacuum, and then, the samples were dried and subsequently surface-modified with PFDTS. The resulting silane-functionalized CNF/MXene (F-MC@SiRF) exhibits a homogeneous black color with a continuous porous structure (Figure 7d), and the thin interconnected MXene/CNF coating bonded to the foam surface can also be seen in the figure with a thickness of 5.2 μm. It was demonstrated by EDS mapping that the hybrid MXene/CNF coatings adhered well to the foam surface. The prepared PDMS composite CNF/MXene conductive foams have tunable electrical conductivity (10^−8^–10 S/m) and superhydrophobicity, stable compression cycling, excellent flame retardancy in the range of −20–200 °C, and can be adapted to a wide range of complex chemical environments. Shi et al. reported a triboelectric nanogenerator (TENG) [82], as shown in Figure 7e, where the composite was dielectrically modulated by BaTiO_3_ and Ag. The above composites were prepared into a porous aerogel using crosslinking, washing, and freeze-drying and finally compressed into cellulose/conducting nanoparticle–PDMS paper. This composite paper has a thickness of 200 μm with excellent flexibility and mechanical robustness. Wang et al. investigated a strategy combining interfacial assembly and encapsulation integration to prepare regenerated cellulose, silver nanowires (AgNWs), and PEDOT:PSS into flexible, transparent, and electrically conductive composite thin films (Figure 7f) [83]. Françon et al. used alginate, CNF, and PEDOT:tosylate (PEDOT:TOS) to prepare composite highly conductive aerogels via a 3D printing technique [84], as shown in Figure 7g. The ratio of alginate to CNF and the ionic strength can precisely control the viscosity of the gels, which allows the printing of thin and stable gel layers. Alginate and CNF can be printed into complex shapes and successively frozen, thawed, solvent-exchanged, and dried into highly porous networks. The conductive aerogel has a minimum density of 23 kg/m^3^ and a high compression modulus of 275 kPa.

In short, researchers have developed various effective strategies to prepare composites of cellulose with various types of conductive substances. Such materials add conductive properties to the sustainability and tunability of cellulose, preparing CCMs for use in energy and sensing.

## 4. Energy Applications

Energy devices are hotly sought after in current research, and it is not only the efficiency of energy storage systems that is now being pursued, but researchers are also showing increasing interest in flexible, stretchable, and wearable energy devices [85,86]. CCMs play an important role in the energy sector due to their rich surface chemistry, unique pore size and network structure, and environmental friendliness. Compared to plastics, cellulose can easily form pore channels for ion and electron transport. Oxygen-containing groups (hydrophilic functional groups) are spread over the surface of the fibers, which have good moisturizing ability in the electrolyte solution, and they have electrostatic repulsion in the solution, which effectively prevents the agglomeration of electrode materials [13,87,88]. In this chapter, the applications of CCMs in batteries and supercapacitors will be introduced, and some cases use cellulose-based ion-conductive materials [11].

### 4.1. Batteries

Cellulose from various biomass sources has an aligned one-dimensional (1D) hierarchical structure consisting of repetitively arranged and dehydrated glucose units that form chains of cellulose molecules with a number of oxygen-containing polar functional groups, such as hydroxyl groups, on the surface [9,10,89]. These polar functional groups can solubilize lithium ions (Li ions) and assist in their movement, which can stabilize the cycling performance of Li batteries and add sustainability through modification or complexation with other advantageous substances [90,91]. In addition, the phonon/electron transport mode of cellulose can be altered by doping with fillers to produce composites with excellent mechanical, thermal, optical, and barrier properties [92]. The three main applications of CCMs in batteries are electrolytes, separators, and battery electrodes. At the end of a battery product’s useful life, cellulose-based components exhibit sustainable properties; they degrade quickly in the natural environment without leaking microplastics, and they do not require additional chemical treatment processes.

**Figure 8:**
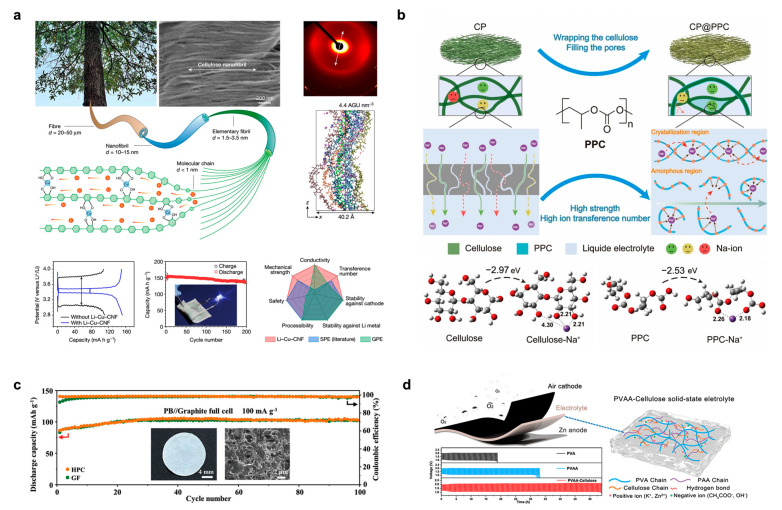
(**a**) Exploration of the evolution of Li-Cu-CNF composite structure and its application in Li-ion batteries. Reprinted with permission from ref. [93]. (**b**) Cellulose-based separator (CP)/poly(propylene carbonate) (CP/PPC) composite with “pore-hopping” ion transport mechanism for Na-ion batteries. Reprinted with permission from ref. [94]. (**c**) Supramolecular and hierarchical porous structures composed of natural cellulose for long-cycle-life K-ion batteries. Reprinted with permission from ref. [95]. (**d**) Ultrafine cellulose/PVA/PAA crosslinked composite gel electrolyte for flexible Zn–air batteries with a long cycle life. Reprinted with permission from ref. [96].

Firstly, the applications of CCMs in Li-ion, sodium-ion (Na-ion), potassium-ion (K-ion), and zinc-ion (Zn-ion) batteries are presented. Yang et al. reported a versatile strategy for the preparation of high-performance cellulose-based solid polymer ionic conductors with molecular channel engineering (Figure 8a), and used in a solid electrolyte/diaphragm for Li-ion batteries [93], the Li-ion batteries are flexible, low-cost, and extensible. The insertion of Cu^2+^ into the CNF structure expands the spacing of the polymer segments directly and allows for rapid Li^+^ doping and transport across the gaps. This Li-Cu-CNF composite structure has a high ionic conductivity of 1.5 × 10^+−3^ S/cm and a transfer number up to 0.78 for Li^+^. It also has a wide voltage window of 0–4.5 V. The pristine CNF has a typical cellulose diffraction pattern, and the diffraction peak position at (200) shows a cellulose molecule chain spacing of 0.39 nm. The tight stacking of this gap prevents Li^+^ from inserting between molecules. In contrast, Cu-CNF is bridged by Cu^2+^ through four coordinated Cu-O bonds, which opens up the tight molecular stacking of CNF to an overall hexagonal crystal structure with an interchain spacing of 0.87 nm. Molecular channels are opened up between the cellulose chains, thereby allowing the insertion of Li^+^ behind these channels. Further, it is demonstrated that Li-Cu-CNF achieves efficient Li^+^ transport in a full cell with a solid LiFePO_4_ cathode. In this cell, Li-Cu-CNF also acts as a binder and exhibits lower impedance, lower overpotential, and higher capacity than conventional materials of this type. The Li-Cu-CNF-based all-solid-state Li-ion battery also achieved 94% capacity retention after 200 cycles. Thus, a stable flexible all-solid-state Li battery consisting of Li-Cu-CNF electrolyte was realized. Yang et al. employed a submerged curing method to laminate cellulose-based separator (CP) and polycarbonate acrylic (PPC) and used them for Na-ion batteries [94]. As shown in Figure 8b, the holes in the special structure of CP@PPC allow the ions to “jump” and promote the ion transfer efficiency, which improves the overall performance of the Na-ion battery. The transfer number of CP@PPC is 0.613, and the whole battery is still 96.97% stable after 500 cycles. Wang et al. reported a natural supramolecular structure which uses the supramolecular and multilevel structure of cellulose for high-performance K-ion batteries [95]. A multi-stage porous cellulose (HPC) separator with an average pore size of 0.64 μm was prepared using CNF, and this natural structure could be utilized to extend the lifetime of the K-ion battery by 1000 h. K-ion batteries assembled from HPC provided a reversible capacity of 86 mAh/g at 100 cycles without capacity degradation (Figure 8c). Li et al. developed an ultrafine cellulose/PVA/PAA crosslinked composite gel electrolyte (PVAA-cellulose) [96], which has an extensive porous network and hydrogen bonding. PVAA-cellulose achieves excellent water retention, thermal stability, and a high ionic conductivity of 123 mS/m, thus enabling the fabrication of a rechargeable solid-state Zn–air battery (Figure 8d). PVAA-cellulose was able to inhibit the growth of Zn dendrites, and the assembled flexible Zn–air battery achieved a high power of 74 mW/cm^2^, a high specific capacity of 724 mAh/g_Zn_, and a cycling stability of 54 h. Zou et al. proposed a composite of BC/gel polymer support membranes and partially amidated intrinsic microporous polymers (PIMs) for Li-ion batteries [97]. A high Li^+^ mobility number of 0.76 and 83% capacity retention (200 cycles) were exhibited in Li-ion batteries.

### 4.2. Supercapacitors

Supercapacitors are energy devices with high power density and high cycling stability because their charge is mainly stored on the surface of the electrodes, so the performance of the electrodes plays a crucial role in supercapacitors [98,99,100]. While binders in conventional electrodes affect the storage of charge or electrochemical substances, the high specific surface area and abundant chemical sites of sustainable cellulose can bind with electrochemically active substances to improve the performance of supercapacitors [101]. Further, cellulose can bring flexibility and excellent mechanical properties to supercapacitors, and the porous structure provides high-speed channels for the diffusion of ions and charges [102,103,104].

**Figure 9:**
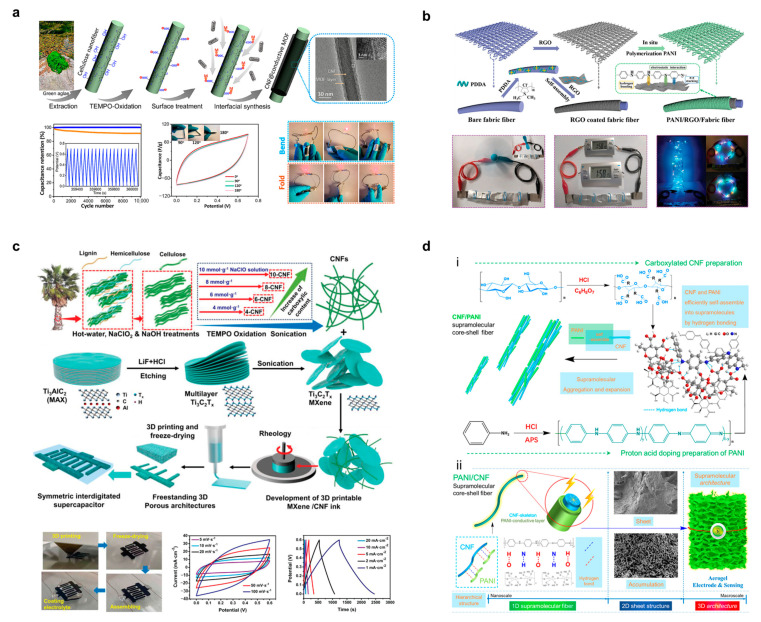
(**a**) Conductive metal–organic framework (c-MOF)/CNF composite electrode for flexible supercapacitors. Reprinted with permission from ref. [105]. (**b**) Fabrication of all-solid-state supercapacitors using RGO/polyester fiber/PANI as flexible electrodes and BC/polyacrylamide as hydrogel electrolyte. Reprinted with permission from ref. [106]. (**c**) Construction of CNF/MXene all-solid-state supercapacitors using 3D printing. Reprinted with permission from ref. [107]. (**d**) Supramolecular self-assembled PANI/CNF conductive aerogel for flexible supercapacitor electrodes. (**i**) stands for supramolecular composites at the molecular level, and (**ii**) represents the hierarchical structure of supramolecular conductive aerogels. Reprinted with permission from ref. [108].

Zhou et al. prepared conductive metal–organic framework (c-MOF)/CNF composite electrodes for flexible supercapacitors using interfacial synthesis [105], as shown in Figure 9a. Combining the high specific surface area of the two, the conductive properties of c-MOF, and the self-crosslinking properties of CNF fabricated paper-like electrodes, which have an electrical conductivity up to 100 S/m, graded pores, and excellent mechanical properties. The capacitance retention of the fabricated supercapacitor was >99% after 10,000 cycles. As shown in Figure 9b, Li et al. fabricated an all-solid-state supercapacitor using RGO-encapsulated polyester fibers and loaded with PANI as flexible electrodes and BC-reinforced polyacrylamide as hydrogel electrolyte [106]. Among them, the textile structure of the flexible electrode was deformable, and the BC-based electrolyte achieved an ionic conductivity of 125 mS/cm. The all-solid-state supercapacitor had a tensile strength of 330 kPa, an elastic tensile ratio of ~1300%, and a high area capacitance of 564 mF/cm^2^, which can power a variety of small electronic devices. Zhou et al. explored the CNF-enhanced MXene rheology and prepared CNF/MXene direct-write inks to construct printable solid-state supercapacitors using 3D printing technology [107], shown in Figure 9c. The supercapacitor has a high area capacitance of 2.02 F/cm^2^, an energy density of 101 μWh/cm^2^, a power density of 0.299 mW/cm^2^, and a capacitance retention rate of 85% after 5000 cycles. Wang et al. discovered a supramolecular self-assembly behavior of PANI and CNF in aqueous medium (Figure 9d) [108] and prepared a composite conductive aerogel with a porosity of 96.90% and a conductivity of 0.372 mS/cm using freeze-drying. A flexible all-solid-state supercapacitor was fabricated using the PANI/CNF composite conductive aerogel as the electrode and PVA/sulfuric acid as the solid-state electrolyte, and the normalized mass capacitance reached 291.01 F/g. For more detailed parameters of the above cellulose-based supercapacitors refer to Table 1.

In short, the application of cellulose in energy devices is blossoming, and various composite structures have been designed for applications with different needs, and the performance is reaching the desired level.

## 5. Sensing Applications

Cellulose has many advantages for the preparation of sensors, such as sustainability, flexibility, texturability, biocompatibility, large specific surface area, and tunable surface chemistry, which are properties that can enhance the response to chemical and biological signals, thus enabling the monitoring of a wide range of analytes at very low concentrations and also the strain response through the physical changes before and after stretching and compression [109,110,111,112]. Additionally, with a rich array of composite strategies, as described in Section 3, CCMs have more opportunities for application in specific sensors.

### 5.1. Chemical and Biological Sensors

Chemical sensors and biosensors are increasingly being used in the monitoring of environmental pollutants (gas) and human pathologies. Unlike traditional large-scale X-ray, spectroscopy, and chromatography equipment, the development of modern portable sensors is moving toward versatility and real-time monitoring, and even some wearable devices have emerged for chemical and biological sensing [113,114,115]. However, these wearable devices are still rigid, do not fit the body well, and are not comfortable. CCMs inherit the flexibility of cellulose, among other features, and play a variety of important roles in the development of chemical and biological sensors.

**Figure 10:**
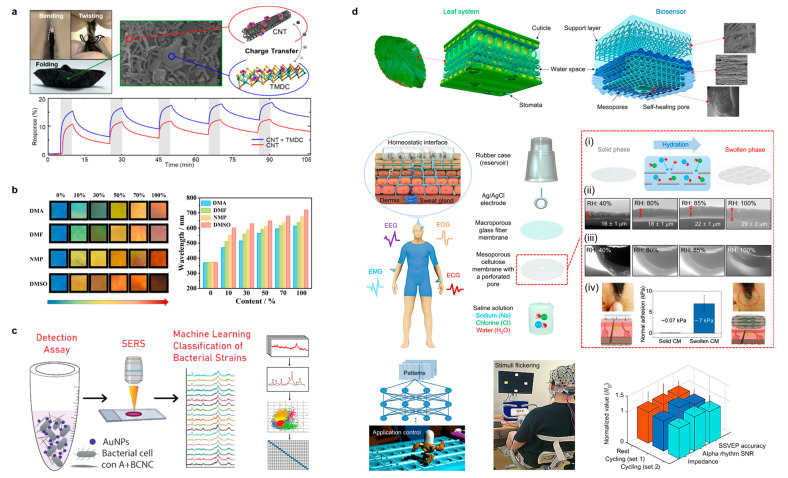
(**a**) Cellulose paper-based chemical sensor for NO_2_ sensing. Reprinted with permission from ref. [116]. (**b**) CNC-based chemical sensor with structured colors to visually display the detected organic solvent species by color. Reprinted with permission from ref. [117]. (**c**) BC-based bacterial sensor that recognizes 19 common bacterial species through SERS and machine learning. The pictures on the right showing the four major steps of developing the SVM predictive model: data collection, preprocessing, model development, and validation/prediction. * represents the finding of characteristic peaks, normalization and baseline correction in mechanical learning. Reprinted with permission from ref. [118]. (**d**) Mesoporous cellulose-based human physiological signaling sensor inspired by natural leaves. Reprinted with permission from ref. [119].

Lee et al. fabricated a sensitive and deformable chemical sensor based on the integration of CNT/WS_2_/MoS_2_ on cellulose paper for sensing [116]. As shown in Figure 10a, the porous composite structure allowed the penetration of multiple chemical gases to obtain an electrical signal response of more than 150% for NO_2_ with a lower response limit of 4.57%/ppm (each ppm change in the gas can cause x% change in the electrical signal response). This cellulose paper-based chemical sensor can be arbitrarily folded and compressed without loss of sensing performance. An interesting CNC-structured color material was proposed by Wang et al. for a chemical sensor, and the unique sensor exhibits different color responses to different organic gases (Figure 10b), enabling one to identify the organic gas species directly with the naked eye with excellent selectivity [117]. The structural color is formed by the copolymerization of CNCs and polydopamine. By controlling the amount of polydopamine, a selective response to organic solvents can be achieved, and a specific color response to different concentrations of single-component organic solvents can also occur. This CNC-based sensor with a color-changing response to chemical reagents pioneers a new detection method that is as convenient as pH test paper. Rahman et al. prepared a biosensor capable of detecting and differentiating among 19 bacterial species by compounding BC with gold nanoparticles (AuNPs) modified with concanavalin A (Figure 10c) [118]. The sensing was realized with surface-enhanced Raman spectroscopy (SERS) combined with machine learning, and the sensing accuracy eventually reached 87.7%. This study combines cellulose-based SERS biosensors with emerging machine learning, expanding an interesting cross-disciplinary direction. Kim et al. received inspiration from the structure of natural leaves and used mesoporous cellulose membranes to fabricate a biosensor for long-term human physiological signal detection [119], as shown in Figure 10d. The whole sensor consists of a salt solution reservoir, Ag/AgCl electrodes, mesoporous cellulose, and stripped cellulose. Mimicking the mechanism of water transport and storage in tree leaves, this mesoporous cellulose membrane-based sensor can permeate the water inside to the skin to increase the fit, and when the skin secretes sweat, it can also allow the sweat to permeate into the sensor in the reverse direction. Through this process, the biosensor can measure an electroencephalogram (EEG), electrooculogram (EOG), electromyography (EMG), and electrocardiogram (ECG) at different locations on the human skin and also incorporates machine learning to display real-time results. The changes in surface morphology and thickness, adhesion, and ionic conductivity of the sensors at different relative humidities were investigated using an environmentally inverted electron cellulose (ESEM). Finally, an individual performing the steady-state visual evoked potentials paradigm while pedaling an exercise bike is shown.

### 5.2. Mechanical Sensor

Cellulosic materials are widely used as substrates for mechanical sensors (such as strain sensors) because of their flexibility and excellent mechanical properties, as well as their sustainability and degradability. CCMs and their compounds can respond to external stress and deformation and output sensing signals through resistive, capacitive, piezoelectric, and friction electric modes, thus realizing the sensing function. Moreover, CCM-based sensors have properties such as portability and wearability and have been used in stretchable sensors and electronic skins for detecting the human body [120,121,122].

**Figure 11:**
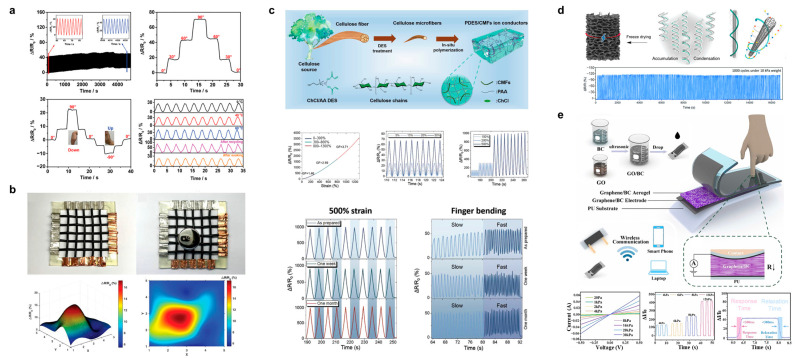
(**a**) Ion conductive strain sensors with double-crosslinked networks for human movement monitoring. Reprinted with permission from ref. [123]. (**b**) Cellulose/graphene/CNT composite e-textile to measure and localize external stress. Reprinted with permission from ref. [124]. (**c**) Cellulose-based ionic conductive conductors for pressure and human motion sensing. Reprinted with permission from ref. [125]. (**d**) CNF/MWCNT composite conductive spring aerogel for long-life surface pressure sensing. Reprinted with permission from ref. [126]. (**e**) GO/BC assembled pressure sensor. Reprinted with permission from ref. [127].

Lu et al. proposed a new strategy of cellulose-derived ion-conductive elastomers for stretchable sensing [123], as shown in Figure 11a, where ion-conductive strain sensors with physically and chemically double-crosslinked networks were prepared using polymerizable deep eutectic solvents. The ΔR/R_0_ values of this strain sensor were confirmed to be stable after ≤1000 cycles with cyclic loading–unloading tests. Further, in the detection of human motion, the ΔR/R_0_ of this sensor responded and was positively correlated with the bending angle and remained stable when the finger was bent to 30°, 60°, and 90° sequentially. In addition, the range of ΔR/R_0_ variation in the detected wrist motion can determine the bending angle of the wrist and also differentiate the direction of wrist downward/upward motion, with a positive response for downward bending of the wrist and a negative response for upward bending. Finally, the stability of the response of this sensor after immersion in organic solvents at low/high temperatures or after recycling is shown. Sheng et al. chose graphene and CNT as a composite conductive filler doped with 2,2,6,6-tetramethylpiperidine-1-oxyl oxidized bacterial cellulose nanofibers (BCNs) and then compounded with thermoplastic polyurethane (TPU). A flexible fiber sensor was prepared with wet spinning for the preparation of wearable devices [124]. Interestingly, as shown in Figure 11b, the fiber sensor can be turned into a 5 × 5 e-textile to measure and localize external stress stimuli. When a 100 g weight is placed on this e-textile, it is easy to observe the position of the object on the 3D surface (color) map constructed by the surf function and the pseudo color. Sun et al. also prepared a cellulose microfiber-based ion-conducting aerogel [125], and deep eutectic solvents can also be used to obtain ion-conducting conductors by polymerizing the two after cellulose extraction (Figure 11c). Tensile strain response tests revealed that the ΔR/R_0_ of this ionic conductor increased monotonically with increasing strain, exhibiting a high response of 3578% at a high strain of 1300%. Additionally, responses at small strains (5%, 10%, 20%, and 50%) and large strains (100%, 200%, and 500%) were recorded, and the ΔR/R_0_ of this sensor showed a highly repeatable and reliable signal. When integrating this cellulose-based strain sensor into the knuckle, it is able to clearly and accurately differentiate bending speeds regardless of storage time, showing potential in future wearable electronics.

Wang et al. proposed a strategy to bend multi-walled CNTs (MWCNTs) into rings using a CNF with a high aspect ratio as a template [126], as shown in Figure 11d, and a composite fiber with a unique chain–ring structure was obtained with a covalent composite of carboxylated CNF and aminated MWCNT. The aerogel prepared with this chain–ring composite fiber has a high compressive resistance of 269.02 kPa and maintains a stable resistive response signal output over 1000 compression cycles. Sun et al. prepared a graphene and BC composite 3D aerogel for pressure sensing [127], where they assembled the composite conductive aerogel/electrode with polyurethane (PU) to form an integrated sensor with wireless transmission protocol to monitor–display the response signal in real time (Figure 11e). Tests showed that this sensor has a good current–voltage (I–V) response in the range of 20 Pa–30 kPa, with different response amplitudes to distinguish the pressure. ΔI/I_0_ dynamic response curves showed good response and recovery behavior at different pressures. The response and recovery delay of the flexible pressure sensor are less than 300 ms at 2 kPa.

In short, the development and application of CCMs in chemical and strain sensors has made effective progress, and the flexibility, sustainability, and molding-assisted capabilities of cellulose are fascinating for applications in wearable sensors. As research progresses, cellulose-based e-textiles are promising to become a commodity.

## 6. Summary and Prospects

The combination of cellulose, a renewable and abundant biopolymer, with a variety of conductive additives (e.g., graphene, CNT, metal particles, MOF, MXene, and conductive polymers, as described in the text) yields materials with excellent electrical conductivity while retaining the inherent biocompatibility and sustainability of cellulose. These materials have been used in a variety of applications, including energy storage, flexible electronics, and sensors. Overall, CCMs have become an important topic of research for energy and sensing applications. This comprehensive review elucidates the remarkable progress made in this field, focusing on the synthesis techniques, properties, and applications of CCMs. Looking ahead, it is clear that these materials have great potential to address the key challenges of sustainable energy and advanced energy and sensing technologies.

In the field of energy storage, CCMs show great promise for applications in batteries and supercapacitors. Their high surface area, tunable porosity, and excellent electrical conductivity contribute to enhanced charge storage capabilities. In addition, the inherent flexibility of cellulose-based materials allows for the development of flexible and lightweight energy storage devices, which are critical for emerging applications in wearable electronics and portable energy. CCMs also make significant contributions to the field of flexible and printed electronics. The ability to process these materials into films, coatings, and inks opens up new avenues for designing flexible and stretchable electronic devices. Such devices can be seamlessly integrated into clothing, skin-like sensors, and Internet of Things (IoT) applications, revolutionizing the way we interact with technology. CCMs offer a unique combination of properties in sensing applications. Their biocompatibility makes them suitable for implantable biosensors and biodegradable environmental sensors. These sensors can be customized to detect a variety of analytes, from sweat to environmental pollutants.

In future, multiple R&D avenues have the potential to further advance CCMs. First, it is important to improve the scalability and cost-effectiveness of synthetic methods and to develop generic paradigm platforms for several types of cellulose-based composites to meet a wide variety of preparation and application needs. Subsequent efforts should be made to improve the mechanical properties and thermal stability of cellulose-based composites to ensure their durability in harsh environments. In addition, exploring new CCM sources and modifications can lead to more desirable material properties. The development of CCMs with synergistic properties remains an exciting avenue for future research.

The problem that none of us can ignore, however, is that it is now impossible to fully explain how nature synthesizes specific cellulose structures, why plants are able to form different cellulose structures in different parts, and why the same all-cellulose skeleton is able to form functionally different specific tissues such as trunks, branches, leaves, grasses, straws, and cell walls. We are still only able to extract cellulose from nature and reuse it; it is possible that one day in the future, researchers will be able to master the formation and regulation mechanisms of cellulose molecules, chain segments, and macrostructures. If this is achieved, this will be the cellulose “big bang”; not limited to conductive applications, cellulose applications will enter the next generation. Then, the manufacturing of cellulose-based conductive materials will not be as monotonous as it is now. Maybe someday, we can prepare integrated cellulose-based batteries or sensors like plants grow naturally. The performance of cellulose-based materials will also be significantly improved, and that will be an era of cellulose materials. In summary, CCMs have made significant progress in energy and sensing applications, providing sustainable and versatile solutions. The continued exploration of synthetic methods, performance enhancements, and novel applications will undoubtedly pave the way for cellulose-based materials to play a key role in shaping the field of sustainable energy and advanced sensing technologies. These materials have the potential to address pressing global challenges and contribute to a more sustainable and technologically advanced future.

## Figures and Tables

**Table 1:** Supercapacitor parameters exhibited by multiple CCM preparation strategies.

Sample	Conductivity	Capacitance	Capacitance Retention	Energy Density	Power Density	Ref.
CNF/c-MOF	100 S/m	103 F/g	>99% after 10,000 cycles	6.5 mWh/cm^2^	0.013 mW/cm^2^	[105]
BC-RGO-PANI	125 mS/cm	564 mF/cm^2^	94.4% after 10,000 cycles	50.1 μWh/cm^2^	20 mW/cm^2^	[106]
CNF/MXene	-	2.02 F/cm^2^	85% after 5000 cycles	101 μWh/cm^2^	0.299 mW/cm^2^	[107]
PANI/CNF	0.372 mS/cm	291.01 F/g	75.6% after 2000 cycles	-	-	[108]

## Data Availability

No new data were created or analyzed in this study.

## Abbreviations

1D	One dimensional
2D	Two dimensional
3D	Three dimensional
BC	Bacterial cellulose
BET	Brunauer–Emmett–Teller
CCMs	Cellulose-based conductive materials
c-MOF	Conductive metal–organic framework
CNCs	Cellulose nanocrystals
CNFs	Cellulose nanofibers
CNFene	CNF-graphene
CNTs	Carbon nanotubes
CP	Cellulose-based separator
DMSO	Dimethyl sulfoxide
ECG	Electrocardiogram
EEG	Electroencephalogram
EMG	Electromyography
EOG	Electrooculogram
FTIR	Fourier-transform infrared
GO	Graphene oxide
MOF	Metal–organic framework
MWCNT	Multi-walled CNT
MXene	Carbides and nitrides of transition metals
NPs	Nanoparticles
PA	Polyacetylene
PAA	Polyacrylic acid
PANI	Polyaniline
PEDOT	Poly(3,4-ethylenedioxythiophene)
PPC	Polycarbonate acrylic
PPy	Polypyrrole
PSS	Polystyrene sulfonate
PT	Polythiophene
PU	Polyurethane
PVA	Polyvinyl alcohol
R&D	Research and development
RGO	Reduced graphene oxide
SEM	Scanning electron microscope
TEMPO	2,2,6,6-tetramethylpiperidine-1-oxylradi-cal
TENG	Triboelectric nanogenerator
TOS	Tosylate
TPU	Thermoplastic polyurethane
XPS	X-ray photoelectron spectroscopy
XRD	X-ray diffraction
